# The Prognostic Value of Calcium in Post-Cardiovascular Surgery Patients in the Intensive Care Unit

**DOI:** 10.3389/fcvm.2021.733528

**Published:** 2021-10-05

**Authors:** Siwei Bi, Ruiqi Liu, Jingyi Li, Shanshan Chen, Jun Gu

**Affiliations:** ^1^West China School of Medicine, Sichuan University, Chengdu, China; ^2^Department of Burn and Plastic Surgery, West China Hospital, Sichuan University, Chengdu, China; ^3^Department of Cardiovascular Surgery, West China Hospital, Sichuan University, Chengdu, China

**Keywords:** cardiovascular surgery, prognosis, calcium, intensive care unit, kidney injury

## Abstract

**Background:** Present researches exploring the prognostic value of calcium concentration are undermined by sample size and study design. Our study investigated the association of both total calcium (tCa) and ionized Ca (iCa) to short- and long-term mortality and other outcomes in post-cardiovascular surgery (PCS) patients admitted to intensive care unit (ICU) from two large public data sets.

**Methods:** The Medical Information Mart for Intensive Care III (MIMIC-III) database and the eICU Collaborative Research Database (eICU) were inspected to identify PCS patients. The primary outcome was 28-day mortality. Multivariate regression was used to elucidate the relationship between calcium concentration and outcomes. The propensity score estimation was performed to validate our findings.

**Results:** A total of 6122 and 914 patients were included from the MIMIC III and eICU data sets, respectively. The groups with the most patients were the mild hypo-iCa and hypo-tCa groups. The mild hypo-iCa group showed significant association with worse short-term and long-term prognosis, less use of ventilation, longer ICU and hospital stay, and more incidence of 7-day acute kidney injury.

**Conclusions:** The mild hypo-iCa (0.9–1.15 mmol/L) within the first day of admission to the ICU could serve as an independent prognosis factor for PCS patients.

## Introduction

Cardiovascular surgery is widely applied for the treatment of cardiovascular disease (1). Post-cardiovascular surgical (PCS) patients have been observed to have unfavorable prognoses and multiple complications such as stroke (2) and acute respiratory distress syndrome (3) resulting from invasive cardiovascular procedures. Another frequently occurred complication, acute kidney injury (AKI), is also associated with increased mortality and morbidity (4) among PCS patients. In severe AKI cases (5), patients would receive continuous renal replacement therapy (CRRT).

Serum calcium is commonly measured among patients in the intensive care unit (ICU) for its significant role in reflecting perturbation of essential physiological processes. Approximately half of serum total calcium (tCa) exists as ionized calcium (iCa), while the rest is combined with proteins or present in the diffusive form (6). The measurement of tCa is favored over iCa for its convenience. Nevertheless, the tCa measure relies highly on protein-bound calcium that is sensitive to the unstable protein level and blood pH among ICU patients. Although correction formulas have been used to adjust tCa with albumin level, their predicting capacity is diminished by various factors (7). Unlike tCa, iCa is unaffected by altered acid-base status or albumin level, thus making it a more reliable parameter in the assessment of patients' calcium status (8) and health status.

There has been emerging evidence regarding the association between tCa, iCa, and the prognosis of ICU patients. Wang et al. (9) demonstrated that for critically ill patients, tCa showed no independent association with mortality, while low iCa levels were important prognosis predictors. In a systematic review investigating hypocalcemia in trauma patients, low iCa at admission was found to be related to elevated mortality (10). Moreover, a retrospective study including 357 pediatric patients established that high iCa level was independently associated with longer ICU stay in pediatric PCS patients (11). The reason for the association between calcium level and patient prognosis could be that calcium takes part in various physiological and biochemical processes such as heart electrophysiology and contraction, blood coagulation, neurotransmitter release, and enzyme activity regulation (12, 13). In severely ill cases, heart failure, and hyperadrenergic states are the most common disorders that are associated with calcium derangements (14). However, due to insufficient sample size and a lack of relevant research, the predictive value of iCa and tCa in the prognosis of ICU PCS patients has not yet been determined.

Therefore, we intended to investigate the association between the calcium concentration (both tCa and iCa) and the important outcomes (short-term and long-term mortality, length of ICU, hospital stay, mechanical ventilation assistance, and AKI in 7 days after the ICU admission) of PCS patients admitted to ICU in a large public clinical database.

## Materials and Methods

### Study Design and Data Source

The study is a retrospective cohort study with data collected from the Medical Information Mart for Intensive Care-III (MIMIC-III) database (15, 16) and the electronic ICU (eICU) (17). Access to the database was approved by the institutional review boards of both Beth Israel Deaconess Medical Center and Massachusetts Institute of Technology Affiliates (authorization code: 40043439). We obtained the anonymous data from the database; therefore, the informed consent was waived. This study is reported following the Strengthening the Reporting of Observational studies in Epidemiology (STROBE) (18) statement.

### Study Population and Outcome

The patients from MIMIC III data set with previous cardiac surgery were identified with current procedural terminology (CPT): the CPT number should be within 33010 and 37799. We included patients whose age was between 18 and 89 years old. If patients were admitted to hospital or ICU multiple times, only the first stay was analyzed. The patients with ICU stay <24 h were excluded. To further improve the credibility of our analysis and results, we also selected PCS patients in eICU database based on the documented treatment information.

**Figure 1:**
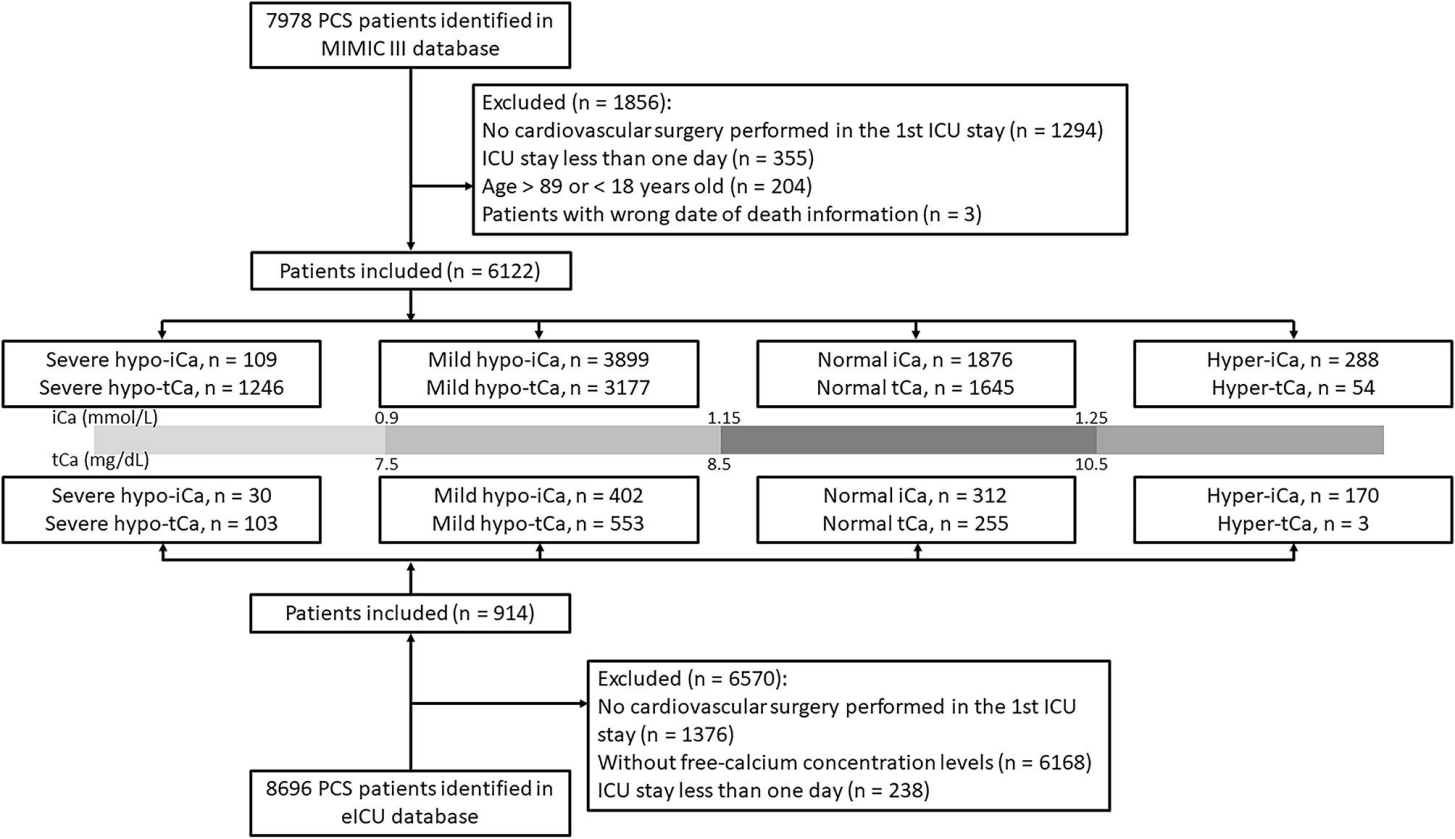
Flow chart of patient selection in two databases.

Based on the concentration of ionized calcium, the study cohort was separated into four groups for further analysis: severe hypo-iCa group (iCa≤ 0.9 mmol/L), mild hypo-iCa group (0.9 < iCa ≤1.15 mmol/L), normal iCa group (1.15 < iCa ≤1.25 mmol/L), and hyper iCa group (iCa > 1.25 mmol/L). We also divided the patients based on the total calcium concentration, which is illustrated in the Figure 1.

In-hospital mortality was chosen as the primary endpoint. Follow-up mortalities, including 28-day mortality, 90-day mortality, and 1-year mortality; the incidence of AKI within 7 days after ICU admission; the length of ventilation, hospital stay, and ICU stay; the use of vasopressor drugs; and the intervention of CRRT within the first day of ICU were the secondary endpoints. The diagnosis of AKI was confirmed following the Kidney Disease: Improving Global Outcomes (KDIGO) guideline (19).

### Data Collection

The data were extracted from the database using structure query language (SQL) with the code in MIMIC Code Repository (https://github.com/MIT-LCP/mimic-website) using PostgreSQL (version 9.4.6, www.postgresql.org). The variables in this study included several parts: (1) demographics; (2) hospitalization and prognosis; (3) scoring systems on ICU admission; (4) vital signs; (5) comorbidities; (6) laboratory results; (7) treatment information including the use of calcium supplement; and (8) surgery type of patients (only obtainable in eICU database). For patients with multiple vital signs and laboratory tests, the mean values within the 24-h after ICU admission were included.

Variables with more than 30% missing values were excluded in the following analyses to avoid potential bias. We completed variables with <30% missing with the multiple imputation method (20).

### Statistical Analysis

Demographics and clinical characteristics were reported using median and standard deviation (SD) for continuous variables and proportions for categorical variables. The Anderson–Darling test was used to determine normality for each continuous variable. Comparisons between groups were made using *t*-tests or Mann–Whitney *U*-test for continuous variables and chi-square or Fisher's exact tests for categorical variables.

Firstly, the LOWESS smooth technique was used to illustrate the relationship between calcium concentration and mortality. We chose the Cox proportional hazards model for the mortality outcomes while the logistics regression model was implemented for other outcomes. Specifically, we conducted the univariate analysis with all selected variables. The ones with significant association with outcomes (*p* < 0.05) and a change in the effect estimate exceeding 10% were adjusted in the following multivariate model.

Moreover, to ensure the robustness of our results, propensity score (PS) estimation (21) was performed to adjust the imbalances between the iCa groups with the TWANG package. The TWANG methods rely on tree-based regression models which are built in an iterative fashion, which were 10,000 in our case. The balance metric contained the max standardized differences between groups with the effect size (ES) and the Kolmogorov–Smirnov (KS) statistic. We controlled the patients on the following variables: age, admission types, marital status, ethnicity, and the use of calcium supplement. Following this, each patient would be assigned a weight and the analysis processes were conducted on the non-matched cohort, and the weighted cohort, separately.

Subsequent subgroup analyses were also performed to further confirm our findings. Patients were divided based on the occurrence of hypertension, diabetes, pulmonary circulation diseases, obesity, AKD in 7 days after ICU admission, use of CRRT or vasopressor, median age, and severity scores. The multivariate cox analysis was performed to investigate the association between calcium groups and 28-day mortality and in-hospital mortality in the MIMIC III data set and eICU data set, respectively.

All data cleaning, statistical analyses and illustration were performed in R software (version 4.0.3) with “tableone” (22), “ggplot2” (23), “survival” (24), “survminer” (25), “lubridate” (26), and “tidyverse” (27). A *p* < 0.05 was considered statistically significant.

## Results

### Baseline Characteristics

The patient selection process is illustrated in Figure 1 and the clinical characteristics at baseline are presented in Supplementary File S1. In the eICU database, a large number of PCS patients were excluded since the absence of free-calcium concentration. The proportion of calcium groups, despite tCa or iCa, was similar in two data sets, and the group with the most patients was the mild hypocalcemia group. The results generated for this group were chosen to be our focus. The in-hospital mortality rate was lower in the eICU (4.3%) data set than the MIMIC III (15.1%) data set.

### The Prognostic Value of iCa and tCa Groups

**Figure 2:**
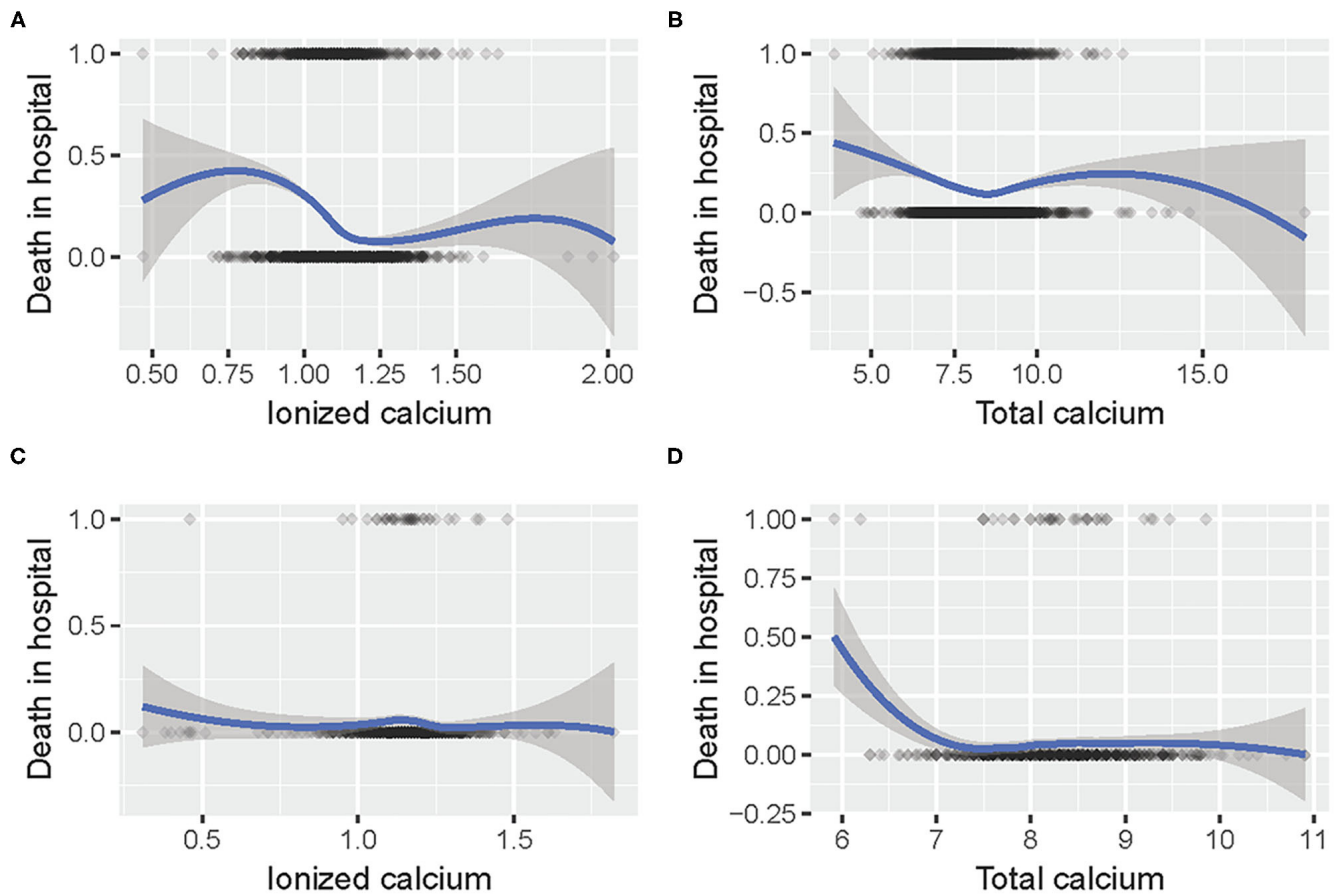
The relationship between **(A)** iCa and mortality in hospital in MIMIC III; **(B)** tCa and mortality in hospital in MIMIC III; **(C)** iCa and mortality in hospital in eICU; **(D)** tCa and mortality in hospital in eICU.

The relationship between calcium concentration and mortality is shown in Figure 2. Given the huge gap between the different numbers of patients, the trends in the two data sets were slightly similar. Moreover, there were some common patterns: the increased mortality rate around 1 mmol/L iCa concentration, and the decreased mortality around 7.5 mg/dl tCa level.

**Figure 3:**
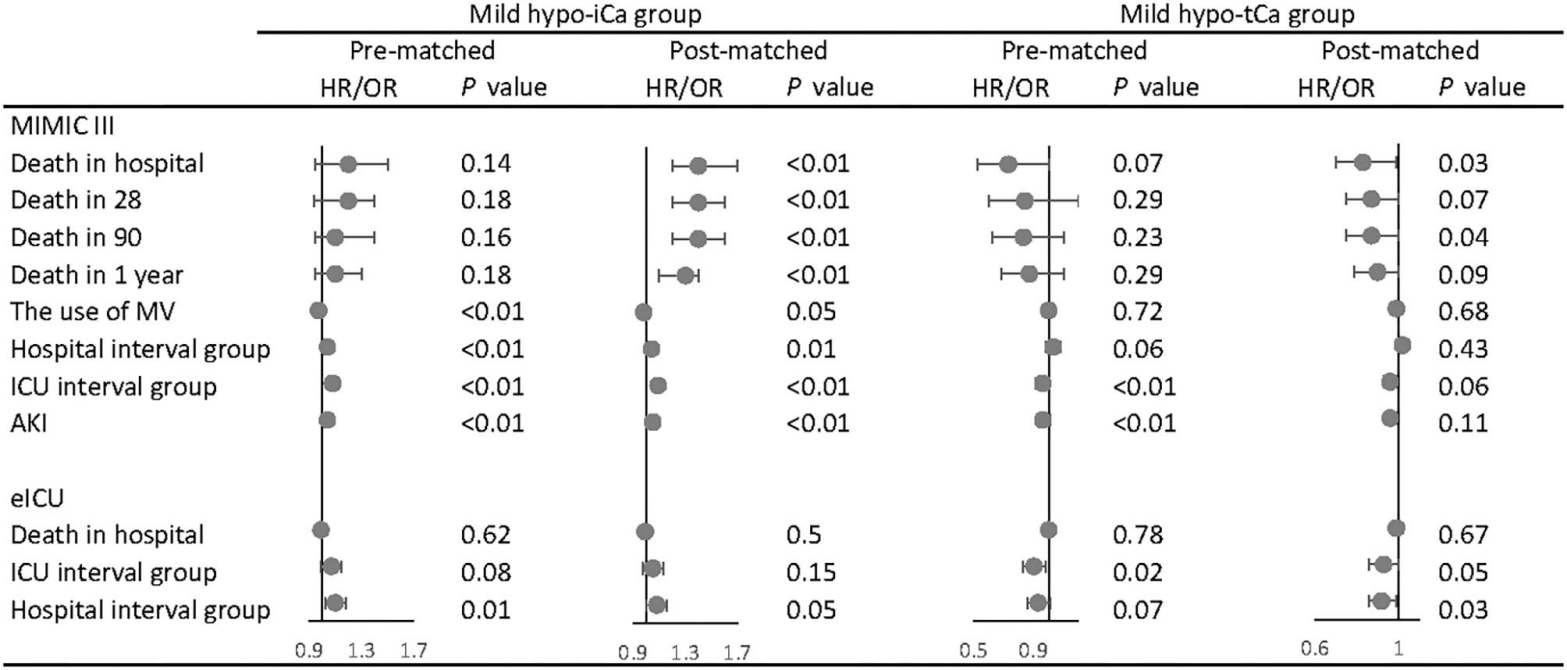
The association between mild hypocalcemia and outcomes. The forest plot represents the hazard ratios (HR) or odds ratios (OR) and 95% confidence intervals (error bars).

Following the aforementioned methods, we calculated the maximum standardized differences between groups (Supplementary Files S2, S8). The results of univariate analysis are attached in Supplementary Files S3, S5, S9, and the multivariate analysis was conducted for both pre-matched and post-matched cohorts (Supplementary Files S4, S6, S10). In the multivariate analysis (Figure 3), the mild hypo-iCa group was proven to be a strong prognosis factor for higher mortality in the pre-and the post-match MIMIC III data set. Patients in the mild hypo-iCa group were less likely to be associated with mechanical ventilation assistance, longer hospital and ICU length, and higher risk of AKD 7 days after discharge from hospital. Similarly, in the eICU data set, the mild hypo-iCa group was also significantly related to the longer hospitalization length (Figure 3).

Although for the tCa groups there was no ample evidence that mild hypo-tCa group was a reliable prognosis factor for mortality, it was associated with less in-hospital mortality as shown in the post-match MIMIC III data set (Figure 3). It had a significant association with shorter hospital interval in eICU data sets (Figure 3).

### Subgroup Analysis

**Figure 4:**
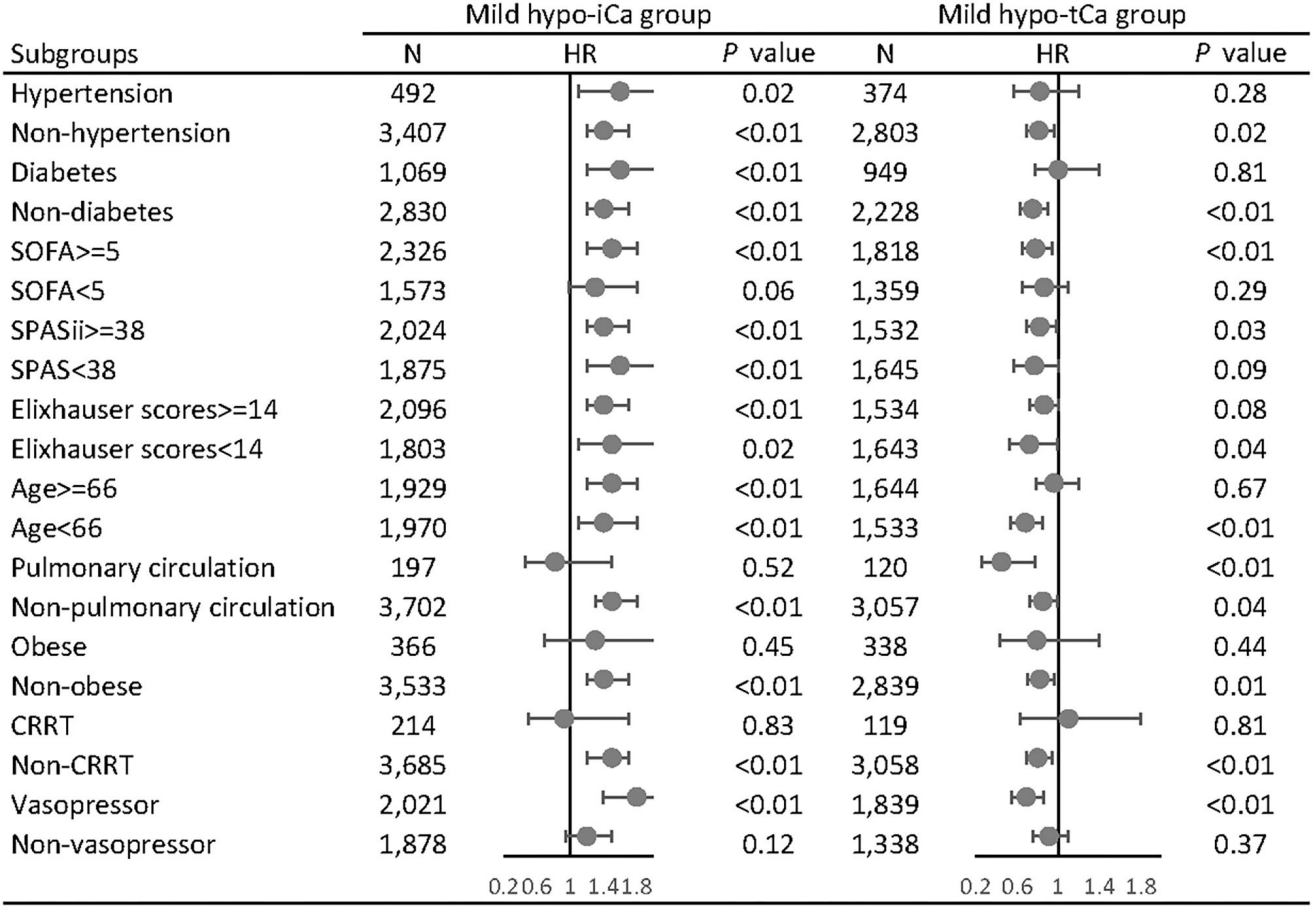
Subgroup analysis of the associations between mild hypocalcemia and 28-day all-cause mortality in MIMIC III database. The forest plot represents the hazard ratios (HR) and 95% confidence intervals (error bars).

In the subgroup analysis, we adjusted the results with gender, age, admission types, marital status, ethnicity, comorbidities, and calcium supplementation in the MIMIC data set and the gender, ethnicity, body mass index (BMI), and calcium supplementation in the eICU data set. In the MIMIC III data set, the mild hypo-iCa group was more likely to be significantly associated with worse prognosis regardless of the disease severity, based on the severity score subgroups (SOFA, SPASii, Elixhauser scores, Figure 4). This trend was not consistent when it came to the results in eICU database (Supplementary File S11).

## Discussion

In the present study, we explored the calcium concentration and the prognosis of PCS patients admitted to ICU. In the MMIC III data set, the mild hypo-iCa group showed significant association with worse short-term and long-term prognosis. The mild-hypo tCa group did not significantly relate with the mortality rate in both cohorts but tended to predict less short-term and long-term mortality. Interestingly, the mild hypo-iCa group was strongly related to less mechanical ventilation use, longer ICU, hospital stay, and more AKI incidence 7 days after discharge.

A previous (28) study focused on the association of iCa with mortality using the MIMIC II database. The moderate hypocalcemia at the ICU admission group (0.8–0.9 mmol/L, 265 patients) was significantly associated with an increased risk of death, while the mild and severe hypocalcemia patients (9,193 and 105 patients, respectively) did not show significant associations. Similarly, various studies had demonstrated the predictive value of lower iCa levels to a worse prognosis (29–31). However, some studies gave contrary results to our study. Steele et al. identified no significant differences between the severe hypocalcemia patients (<0.9 mmol/L) and the normal patients (32). Egi et al. (33) also found iCa concentration had no independent association with hospital or ICU mortality except for the extreme abnormalities. As for the calcium concentration for cardiovascular disease patients, three studies with China patients reported that lower baseline serum calcium levels were associated with a higher risk of in-hospital mortality in myocardial infarction patients (34–36). In another German study, calcium was strongly associated with all-cause mortality but not with cardiovascular events among stable coronary disease patients (37).

The reasons for differing results could be either the study patients (disease severity, number of patients, etc.), the single-center study design, the time point of sample collection, or the calcium supplementation intervention. It should also be noted that there was no consensus on the management of iCa disorder in ICU. The present study firstly implemented PS estimation method to adjust the imbalances between the iCa groups in PCS patients admitted to ICU. The results indicated that the mild hypo-iCa group significantly related to the worse prognosis outcomes (hospital, 28-day, 90-day, and 1-year mortality) in both pre-and post- matched MIMIC III data set but not significant in the eICU data set (in-hospital mortality). This was also a reflection of how results would vary since the heterogeneity of patients and institutions. Further novel-designed researches about the predictive effect of hypo-iCa are still required for more solid conclusions.

AKI is commonly caused by acute glomerular renal diseases, ischemia injury, and acute post-renal obstructive nephropathy (38). The association between calcium and kidney dysfunction could be explained by the vital role of calcium played in many biological processes, e.g., enzyme activity, vasodilatation, inflammation, thrombosis, and vascular smooth muscle movement (39, 40). Studies also suggested that hypocalcemia may alter the cardiac action potential and reduce renal sodium excretion, leading to fluid overload and decreased ventricular work index (35). In a recently published paper, Wang et al. (41) assessed the association of admission tCa and iCa with all-cause mortality in patients with AKI. They concluded low-iCa group (<1.06 mmol/L) was an independent predictor of all-cause mortality (30-, 90-, and 365-day) in critically ill patients with AKI. However, they did not stratify patients accurately enough and did not use the PS estimation method. Our findings suggested that the mild hypo-iCa group had an independent association with more AKI incidence 7 days after discharge for PCS patients, while tCa only significantly associated less incidence in the pre-match cohort. Moreover, for PCS patients with CRRT treatment, both iCa and tCa groups failed to related to the prognosis. The reason might be the disturbances or inaccurate measurement of calcium concentration caused by CRRT (42).

The strengths of our study are obvious. The present study is based on two well-established public databases and a comprehensive analysis process. A large number of patients and plenty of outcomes and results are available for further research and discussion. There are also several limitations in our study. Firstly, the detailed calcium measurement protocol is not clear in both MIMIC III and eICU databases, and the calcium level is actually a time-varying variable. Taking the mean value would cause the loss of certain information. Such time-varying variables can be analyzed with the Cox regression model to estimate its effect on survival time in future research (43). Secondly, the number of cases with extreme calcium values is relatively smaller compared with the mild group. Additionally, the PCS patients received different surgery types, which brings heterogeneity. Our study is a retrospective study, which means the causation cannot be established and only limited information about patients is available. There are several non-negligible contributors to the calcium level such as parathyroid hormone (PTH), calcitonin, and calcitriol, which were not included in the analysis.

## Conclusions

To sum up, the mild hypo-iCa concentration (0.9–1.15 mmol/L) within the first 24 h of ICU admission is associated with a higher risk of short- and long-term mortality, less use of mechanical ventilation, longer ICU and hospital stay, and the AKI 7 days after discharge in PCS patients admitted to ICU. Clinicians should be cautious if patients' iCa concentration falls into this range. Further studies are still needed to determine the actual role of ionized and total calcium levels in disease states and the underlying mechanisms.

## Data Availability Statement

Publicly available datasets were analyzed in this study. This data can be found here: https://eicu-crd.mit.edu/ and https://mimic.mit.edu/.

## Author Contributions

SB, RL, and JL collected, analyzed, and interpreted the data. SB, RL, and SC contributed in writing the manuscript. JL and SC prepared the results. JG generated the idea, provided funding, and revised the manuscript. All authors read and approved the final manuscript.

## Funding

This current project was supported by the National Natural Science Foundation of China (Grant No. 81700410), the Sichuan Science and Technology Program, China (Grant Nos. 2019YFS0344, 2019YFS0251, and 2019YFS0352), and Post-Doctor Research Project, West China Hospital, Sichuan University (20HXBH171).

## Conflict of Interest

The authors declare that the research was conducted in the absence of any commercial or financial relationships that could be construed as a potential conflict of interest.

## Publisher's Note

All claims expressed in this article are solely those of the authors and do not necessarily represent those of their affiliated organizations, or those of the publisher, the editors and the reviewers. Any product that may be evaluated in this article, or claim that may be made by its manufacturer, is not guaranteed or endorsed by the publisher.
