# Supplementary material for: The Prognostic Value of Calcium in Post-Cardiovascular Surgery Patients in the Intensive Care Unit
Source: Front Cardiovasc Med. 2021 Oct 5;8:733528. doi: 10.3389/fcvm.2021.733528 (PMC8523822; doi:10.3389/fcvm.2021.733528)
Supplement: Supplementary file 1 [file Table_1.DOCX]

Table 1. Comparisons of baseline characteristics between the iCa groups in two databases.

|  | MIMIC III | | | | |  | eICU | | | | |  |
| --- | --- | --- | --- | --- | --- | --- | --- | --- | --- | --- | --- | --- |
|  | Overall | Normal | Severe hypo-iCa | Mild hypo-iCa | Hyper-iCa | P value | Overall | Normal | Severe hypo-iCa | Mild hypo-iCa | Hyper-iCa | P value |
| Number of patients | 6122 | 1834 | 120 | 3874 | 294 |  | 914 | 312 | 30 | 402 | 170 |  |
| Gender: Male, n (%) | 3704 (60.5) | 1104 (60.2) | 66 (55.0) | 2377 (61.4) | 157 (53.4) | 0.03 | 606 (66.3) | 199 (63.8) | 21 (70.0) | 270 (67.2) | 116 (68.2) | 0.68 |
| Age, mean (SD) | 64.3 (14.9) | 66.0 (13.8) | 57.2 (16.2) | 63.6 (15.3) | 65.9 (14.5) | <0.01 | 66.0 (13.1) | 66.8 (12.0) | 60.6 (14.8) | 66.0 (13.8) | 65.8 (12.7) | 0.10 |
| Admission type, n (%) |  |  |  |  |  | <0.01 |  |  |  |  |  |  |
| Elective | 1535 (25.1) | 670 (36.5) | 3 (2.5) | 775 (20.0) | 87 (29.6) |  | - | - | - | - | - |  |
| Emergency | 4487 (73.3) | 1143 (62.3) | 112 (93.3) | 3029 (78.2) | 203 (69.0) |  | - | - | - | - | - |  |
| Urgent | 100 (1.6) | 21 (1.1) | 5 (4.2) | 70 (1.8) | 4 (1.4) |  | - | - | - | - | - |  |
| Marital status, n (%) |  |  |  |  |  | <0.01 |  |  |  |  |  |  |
| Married | 3175 (51.9) | 994 (54.2) | 53 (44.2) | 1977 (51.0) | 151 (51.4) |  | - | - | - | - | - |  |
| Notspecified | 352 (5.7) | 63 (3.4) | 10 (8.3) | 266 (6.9) | 13 (4.4) |  | - | - | - | - | - |  |
| Single | 2595 (42.4) | 777 (42.4) | 57 (47.5) | 1631 (42.1) | 130 (44.2) |  | - | - | - | - | - |  |
| Ethnicity, n (%) |  |  |  |  |  | 0.01 |  |  |  |  |  | 0.02 |
| Asian | 130 (2.1) | 27 (1.5) | 3 (2.5) | 98 (2.5) | 2 (0.7) |  | 123 (13.5) | 45 (14.4) | 2 (6.7) | 53 (13.2) | 23 (13.5) |  |
| Black | 377 (6.2) | 107 (5.8) | 15 (12.5) | 232 (6.0) | 23 (7.8) |  | 17 (1.9) | 8 (2.6) | 1 (3.3) | 7 (1.7) | 1 (0.6) |  |
| Hispanic | 179 (2.9) | 54 (2.9) | 5 (4.2) | 111 (2.9) | 9 (3.1) |  | 714 (78.1) | 244 (78.2) | 20 (66.7) | 309 (76.9) | 141 (82.9) |  |
| Not specified | 1004 (16.4) | 281 (15.3) | 26 (21.7) | 647 (16.7) | 50 (17.0) |  | 10 (1.1) | 2 (0.6) | 1 (3.3) | 6 (1.5) | 1 (0.6) |  |
| White | 4432 (72.4) | 1365 (74.4) | 71 (59.2) | 2786 (71.9) | 210 (71.4) |  | 50 (5.5) | 13 (4.2) | 6 (20.0) | 27 (6.7) | 4 (2.4) |  |
| Hospitalization and prognosis, mean (SD) |  |  |  |  |  |  |  |  |  |  |  |  |
| ICU interval, day | 6.9 (9.1) | 5.2 (7.2) | 12.1 (12.6) | 7.6 (9.6) | 5.6 (9.3) | <0.01 | 535 (58.5) | 176 (56.4) | 19 (63.3) | 252 (62.7) | 88 (51.8) | 0.07 |
| Hospital interval, day | 14.4 (14.0) | 11.6 (10.0) | 19.7 (19.4) | 15.7 (15.3) | 12.8 (12.3) | <0.01 | 509 (55.7) | 168 (53.8) | 16 (53.3) | 247 (61.4) | 78 (45.9) | 0.01 |
| Survival time, day | 108.9 (331.8) | 99.3 (330.0) | 88.0 (249.1) | 113.3 (334.3) | 118.8 (336.1) | 0.41 | - | - | - | - | - |  |
| Scoring system, mean (SD) |  |  |  |  |  |  |  |  |  |  |  |  |
| SOFA | 5.7 (3.5) | 5.2 (2.9) | 8.6 (4.8) | 5.8 (3.6) | 6.0 (3.4) | <0.01 | 3.9 (2.5) | 3.8 (2.4) | 4.1 (3.2) | 4.0 (2.5) | 3.9 (2.2) | 0.88 |
| SPAS II | 40.0 (14.3) | 38.2 (12.9) | 50.4 (18.4) | 40.4 (14.5) | 41.7 (14.6) | <0.01 | - | - | - | - | - |  |
| Elixhauser scores | 15.2 (13.8) | 13.1 (13.1) | 22.1 (14.7) | 16.0 (14.0) | 15.1 (13.5) | <0.01 | - | - | - | - | - |  |
| Apache iv | - | - | - | - | - |  | 58.2 (23.9) | 57.4 (25.3) | 53.0 (25.5) | 57.9 (23.6) | 61.1 (21.6) | 0.22 |
| Vital signs, mean (SD) |  |  |  |  |  |  |  |  |  |  |  |  |
| BMI | 50.6 (597.8) | 53.2 (571.8) | 28.0 (7.7) | 50.4 (634.8) | 47.0 (306.2) | 0.97 | 28.3 (5.1) | 28.7 (5.5) | 27.5 (4.3) | 27.7 (4.9) | 28.9 (4.8) | 0.02 |
| Heart rate mean, beats/min | 87.7 (15.3) | 85.3 (13.1) | 98.7 (19.7) | 88.6 (15.9) | 86.4 (14.4) | <0.01 | 83.3 (13.2) | 83.2 (13.9) | 78.9 (14.8) | 83.6 (13.1) | 83.5 (11.4) | 0.31 |
| Systolic BP mean, mmHg | 115.3 (14.5) | 114.7 (12.7) | 116.1 (19.9) | 115.6 (15.2) | 115.3 (12.9) | 0.21 | - | - | - | - | - |  |
| Diastolic BP mean, mmHg | 59.4 (9.6) | 58.2 (8.5) | 62.9 (12.6) | 59.9 (10.0) | 58.3 (9.0) | <0.01 | - | - | - | - | - |  |
| Mean BP mean, mmHg | 76.5 (9.8) | 75.7 (8.5) | 78.6 (13.8) | 76.9 (10.2) | 75.7 (9.0) | <0.01 | - | - | - | - | - |  |
| Respiratory rate mean, /min | 19.0 (4.1) | 18.1 (3.6) | 22.3 (4.8) | 19.4 (4.3) | 18.2 (3.6) | <0.01 | 18.1 (3.5) | 18.1 (3.7) | 18.9 (3.0) | 18.1 (3.3) | 18.0 (3.4) | 0.63 |
| Temperature mean, celcius | 36.9 (0.7) | 36.8 (0.6) | 36.9 (0.9) | 36.9 (0.7) | 36.7 (0.6) | <0.01 | 36.9 (0.6) | 36.9 (0.6) | 36.9 (0.5) | 36.9 (0.6) | 37.0 (0.5) | 0.04 |
| SpO2 mean, % | 97.4 (2.1) | 97.7 (1.6) | 96.2 (3.4) | 97.3 (2.2) | 97.5 (2.3) | <0.01 | 97.4 (1.7) | 97.4 (1.7) | 97.5 (1.8) | 97.4 (1.8) | 97.6 (1.6) | 0.75 |
| PaO2 mean, mmHg | 172.4 (70.1) | 191.8 (69.8) | 134.0 (61.9) | 163.1 (67.9) | 189.7 (72.1) | <0.01 | 146.2 (70.7) | 145.4 (72.7) | 120.9 (40.3) | 152.0 (73.6) | 138.4 (62.7) | 0.03 |
| PaCO2 mean, mmHg | 41.0 (8.3) | 42.1 (7.7) | 35.8 (8.5) | 40.5 (8.4) | 42.2 (8.1) | <0.01 | 41.0 (5.6) | 41.5 (5.8) | 40.4 (5.1) | 40.6 (5.7) | 41.5 (4.7) | 0.13 |
| pH mean | 7.4 (0.6) | 7.4 (0.1) | 7.3 (0.1) | 7.4 (0.1) | 7.5 (2.7) | <0.01 | 7.4 (0.1) | 7.4 (0.1) | 7.4 (0.1) | 7.4 (0.1) | 7.4 (0.0) | 0.42 |
| Comorbidities, n (%) |  |  |  |  |  |  |  |  |  |  |  |  |
| Congestive heart failure | 816 (13.3) | 180 (9.8) | 28 (23.3) | 571 (14.7) | 37 (12.6) | <0.01 | - | - | - | - | - |  |
| Cardiac arrhythmias | 911 (14.9) | 190 (10.4) | 24 (20.0) | 669 (17.3) | 28 (9.5) | <0.01 | - | - | - | - | - |  |
| Valvular disease | 254 (4.1) | 60 (3.3) | 6 (5.0) | 176 (4.5) | 12 (4.1) | 0.15 | - | - | - | - | - |  |
| Pulmonary circulation | 258 (4.2) | 55 (3.0) | 11 (9.2) | 184 (4.7) | 8 (2.7) | <0.01 | - | - | - | - | - |  |
| Hypertension | 758 (12.4) | 213 (11.6) | 15 (12.5) | 474 (12.2) | 56 (19.0) | <0.01 | - | - | - | - | - |  |
| Chronic pulmonary | 206 (3.4) | 55 (3.0) | 2 (1.7) | 143 (3.7) | 6 (2.0) | 0.20 | - | - | - | - | - |  |
| Hypothyroidism | 1179 (19.3) | 378 (20.6) | 15 (12.5) | 728 (18.8) | 58 (19.7) | 0.10 | - | - | - | - | - |  |
| Renal failure | 869 (14.2) | 247 (13.5) | 19 (15.8) | 545 (14.1) | 58 (19.7) | 0.04 | - | - | - | - | - |  |
| Liver disease | 463 (7.6) | 97 (5.3) | 13 (10.8) | 330 (8.5) | 23 (7.8) | <0.01 | - | - | - | - | - |  |
| Rheumatoid arthritis | 182 (3.0) | 52 (2.8) | 5 (4.2) | 118 (3.0) | 7 (2.4) | 0.77 | - | - | - | - | - |  |
| Coagulopathy | 1153 (18.8) | 267 (14.6) | 52 (43.3) | 785 (20.3) | 49 (16.7) | <0.01 | - | - | - | - | - |  |
| Obesity | 612 (10.0) | 207 (11.3) | 3 (2.5) | 368 (9.5) | 34 (11.6) | 0.01 | - | - | - | - | - |  |
| Diabetes | 1827 (29.8) | 610 (33.3) | 29 (24.2) | 1066 (27.5) | 122 (41.5) | <0.01 | - | - | - | - | - |  |
| Cancer | 445 (7.3) | 109 (5.9) | 12 (10.0) | 310 (8.0) | 14 (4.8) | 0.01 | - | - | - | - | - |  |
| Laboratory results, mean (SD) |  |  |  |  |  |  |  |  |  |  |  |  |
| Anion gap, mEq/L | 13.1 (3.9) | 11.9 (2.9) | 19.2 (6.8) | 13.6 (3.9) | 12.5 (3.3) | <0.01 | 9.1 (3.8) | 9.0 (3.8) | 8.6 (4.2) | 9.4 (3.8) | 8.7 (3.3) | 0.11 |
| Bicarbonate, mEq/L | 23.1 (4.0) | 23.8 (3.4) | 19.0 (5.2) | 22.9 (4.2) | 23.6 (3.6) | <0.01 | 23.7 (2.8) | 23.9 (3.0) | 24.4 (3.4) | 23.6 (2.8) | 23.7 (2.4) | 0.26 |
| Creatine Kinase MB Isoenzyme, ng/mL | 1.4 (1.5) | 1.2 (1.1) | 3.3 (3.4) | 1.5 (1.5) | 1.5 (1.6) | <0.01 | 1.2 (1.0) | 1.1 (0.8) | 1.1 (0.4) | 1.3 (1.3) | 1.1 (0.6) | 0.02 |
| Chloride, mmol/L | 106.7 (5.4) | 107.3 (4.4) | 104.6 (7.9) | 106.4 (5.7) | 107.8 (4.9) | <0.01 | 106.6 (4.4) | 106.4 (4.6) | 107.3 (4.5) | 106.8 (4.5) | 106.4 (4.1) | 0.42 |
| glucose | 139.1 (43.2) | 134.5 (31.1) | 154.4 (71.3) | 140.7 (46.8) | 140.3 (39.6) | <0.01 | 135.6 (25.9) | 135.9 (24.0) | 139.2 (35.7) | 134.8 (28.5) | 136.1 (20.2) | 0.78 |
| Hematocrit, % | 30.8 (4.9) | 30.5 (4.6) | 31.1 (5.9) | 30.9 (5.0) | 30.0 (4.6) | <0.01 | 31.2 (4.9) | 31.4 (5.1) | 31.3 (5.2) | 31.1 (4.8) | 31.0 (4.8) | 0.82 |
| Hemoglobin, g/dL | 10.4 (1.7) | 10.3 (1.6) | 10.6 (2.0) | 10.4 (1.8) | 10.0 (1.6) | <0.01 | 10.5 (1.7) | 10.6 (1.7) | 10.5 (1.7) | 10.5 (1.6) | 10.4 (1.6) | 0.81 |
| Lactate, mmol/L | 2.4 (1.6) | 2.2 (1.1) | 3.6 (3.2) | 2.4 (1.7) | 2.5 (1.6) | <0.01 | - | - | - | - | - |  |
| Platelet count, 10^9^/L | 192.8 (99.0) | 191.4 (88.0) | 170.8 (120.3) | 194.5 (102.8) | 186.5 (103.1) | 0.03 | 165.1 (71.8) | 170.3 (72.4) | 169.6 (53.9) | 161.4 (76.3) | 163.6 (61.5) | 0.40 |
| Potassium, mmol/L | 4.2 (0.5) | 4.3 (0.5) | 4.1 (0.8) | 4.2 (0.5) | 4.4 (0.6) | <0.01 | 4.3 (0.4) | 4.2 (0.4) | 4.2 (0.4) | 4.2 (0.5) | 4.4 (0.4) | 0.01 |
| PTT | 38.4 (16.8) | 37.0 (14.9) | 41.9 (19.1) | 39.0 (17.5) | 38.7 (16.5) | <0.01 | - | - | - | - | - |  |
| INR | 1.5 (0.7) | 1.4 (0.5) | 1.9 (1.7) | 1.5 (0.7) | 1.4 (0.7) | <0.01 | - | - | - | - | - |  |
| PT | 15.8 (4.9) | 15.2 (3.6) | 18.2 (7.6) | 16.0 (5.2) | 15.9 (5.7) | <0.01 | - | - | - | - | - |  |
| Sodium, mmol/L | 138.1 (4.1) | 138.0 (3.5) | 138.4 (5.6) | 138.1 (4.3) | 138.6 (4.5) | 0.14 | 138.7 (3.6) | 138.7 (3.9) | 139.2 (3.5) | 138.8 (3.5) | 138.3 (3.3) | 0.28 |
| BUN, mg/dL | 25.5 (21.1) | 21.7 (14.7) | 50.0 (37.4) | 26.4 (22.4) | 26.5 (19.3) | <0.01 | 19.8 (10.8) | 19.6 (10.2) | 19.0 (9.9) | 20.1 (11.7) | 19.8 (9.6) | 0.89 |
| White blood cells, 10^9^/L | 13.0 (8.6) | 13.0 (6.1) | 11.5 (7.1) | 13.1 (9.8) | 13.0 (6.0) | 0.27 | 12.8 (4.9) | 13.4 (5.1) | 11.4 (4.5) | 12.3 (4.6) | 13.3 (4.9) | 0.01 |
| Treatment |  |  |  |  |  |  |  |  |  |  |  |  |
| Vasopressor, % | 3417 (55.8) | 1171 (63.8) | 50 (41.7) | 2012 (51.9) | 184 (62.6) | <0.01 | - | - | - | - | - |  |
| CRRT, % | 299 (4.9) | 51 (2.8) | 28 (23.3) | 204 (5.3) | 16 (5.4) | <0.01 | - | - | - | - | - |  |
| Use of mechanical ventilation | 5094 (83.2) | 1611 (87.8) | 92 (76.7) | 3143 (81.1) | 248 (84.4) | <0.01 | - | - | - | - | - |  |
| Calcium supplement | 4420 (72.2) | 1302 (71.0) | 106 (88.3) | 2806 (72.4) | 206 (70.1) | <0.01 | 73 (8.0) | 17 (5.4) | 6 (20.0) | 40 (10.0) | 10 (5.9) | 0.01 |
| Surgery type |  |  |  |  |  |  |  |  |  |  |  | <0.01 |
| Aneurysm | - | - | - | - | - |  | 64 (7.0) | 25 (8.0) | 2 (6.7) | 25 (6.2) | 12 (7.1) |  |
| CABG | - | - | - | - | - |  | 384 (42.0) | 138 (44.2) | 11 (36.7) | 148 (36.8) | 87 (51.2) |  |
| CABG and valve | - | - | - | - | - |  | 34 (3.7) | 8 (2.6) | 2 (6.7) | 14 (3.5) | 10 (5.9) |  |
| Implantation | - | - | - | - | - |  | 41 (4.5) | 16 (5.1) | 3 (10.0) | 18 (4.5) | 4 (2.4) |  |
| Other vascular bypass | - | - | - | - | - |  | 45 (4.9) | 15 (4.8) | 0 (0.0) | 28 (7.0) | 2 (1.2) |  |
| Others | - | - | - | - | - |  | 45 (4.9) | 16 (5.1) | 5 (16.7) | 21 (5.2) | 3 (1.8) |  |
| Thrombectomy | - | - | - | - | - |  | 35 (3.8) | 14 (4.5) | 3 (10.0) | 14 (3.5) | 4 (2.4) |  |
| Valve | - | - | - | - | - |  | 266 (29.1) | 80 (25.6) | 4 (13.3) | 134 (33.3) | 48 (28.2) |  |
| Outcomes, n (%) |  |  |  |  |  |  |  |  |  |  |  |  |
| Death in hospital | 925 (15.1) | 164 (8.9) | 42 (35.0) | 680 (17.6) | 39 (13.3) | <0.01 | 39 (4.3) | 16 (5.1) | 1 (3.3) | 17 (4.2) | 5 (2.9) | 0.71 |
| Death in 28 days | 1114 (18.2) | 203 (11.1) | 44 (36.7) | 818 (21.1) | 49 (16.7) | <0.01 | - | - | - | - | - |  |
| Death in 90 days | 1301 (21.3) | 250 (13.6) | 46 (38.3) | 949 (24.5) | 56 (19.0) | <0.01 | - | - | - | - | - |  |
| Death in 1 year | 1606 (26.2) | 335 (18.3) | 51 (42.5) | 1147 (29.6) | 73 (24.8) | <0.01 | - | - | - | - | - |  |
| Longer hospital interval group | 3056 (49.9) | 735 (40.1) | 92 (76.7) | 2103 (54.3) | 126 (42.9) | <0.01 | 509 (55.7) | 168 (53.8) | 16 (53.3) | 247 (61.4) | 78 (45.9) | 0.01 |
| Longer ICU interval group | 3056 (49.9) | 707 (38.5) | 88 (73.3) | 2143 (55.3) | 118 (40.1) | <0.01 | 535 (58.5) | 176 (56.4) | 19 (63.3) | 252 (62.7) | 88 (51.8) | 0.07 |
| AKI in 7 days | 4563 (74.5) | 1274 (69.5) | 110 (91.7) | 2967 (76.6) | 212 (72.1) | <0.01 | - | - | - | - | - |  |
| Calcium risk group, n (%) |  |  |  |  |  | <0.01 |  |  |  |  |  | 0.01 |
| Normal | 1636 (26.7) | 714 (38.9) | 6 (5.0) | 722 (18.6) | 194 (66.0) |  | 254 (27.8) | 82 (26.3) | 15 (50.0) | 104 (25.9) | 53 (31.2) |  |
| Severe hypo | 1198 (19.6) | 111 (6.1) | 87 (72.5) | 992 (25.6) | 8 (2.7) |  | 112 (12.3) | 34 (10.9) | 3 (10.0) | 64 (15.9) | 11 (6.5) |  |
| Mild hypo | 3237 (52.9) | 996 (54.3) | 27 (22.5) | 2151 (55.5) | 63 (21.4) |  | 545 (59.6) | 195 (62.5) | 12 (40.0) | 233 (58.0) | 105 (61.8) |  |
| Hyper | 51 (0.8) | 13 (0.7) | 0 (0.0) | 9 (0.2) | 29 (9.9) |  | 3 (0.3) | 1 (0.3) | 0 (0.0) | 1 (0.2) | 1 (0.6) |  |

MIMIC III: Medical Information Mart for Intensive Care-III; eICU: the eICU Collaborative Research Database; SOFA: sequential organ failure assessment; SPAS: simplified acute physiology score; BMI: body mass index; PTT: partial thromboplastin time; PT: Prothrombin Time; INR: international normalized ratio; CRRT: continuous renal replacement therapy; CABG: coronary artery bypass graft; AKI: acute kidney injury; ICU: the intensive care unit; CK-MB: creatine kinase myocardial band; BUN: blood urea nitrogen.
